# 295 Topical Tranexamic Acid in Burn Surgery – A Randomized Controlled Trial

**DOI:** 10.1093/jbcr/irad045.270

**Published:** 2023-08-29

**Authors:** Robert Norheim Colclough, Stian Kreken Almeland, Kjersti Ausen, Ragnvald Ljones L Brekke, Håvard Nordgaard, Hilde Pleym, Olav Spigset

**Affiliations:** Norwegian National Burn Center, Haukeland University Hospital, Bergen, Hordaland; Haukeland University Hospital, Bergen, Hordaland; St. Olav’s University Hospital, Trondheim, Trondheim, Sor-Trondelag; Haukeland University Hospital, Nesttun, Hordaland; St. Olav’s University Hospital, Trondheim, Trondheim, Sor-Trondelag; St. Olav’s University Hospital, Trondheim, Trondheim, Sor-Trondelag; St. Olav’s University Hospital, Trondheim, Sor-Trondelag

## Abstract

**Introduction:**

Early excision and grafting of large burn wounds can result in considerable bleeding. Systemic use of tranexamic acid (TXA) generally reduces surgical bleeding by 30-40%, but its safety in burn surgery has not been studied. Topical use may provide a sufficient wound concentration with negligible risk of systemic adverse events. However, it is unclear whether topical use may delay wound re-epithelialization. This pilot double-blinded randomized controlled trial aimed to evaluate the therapeutic potential of topical TXA for reducing blood loss and the effect on wound re-epithelialization in burn surgery.

**Methods:**

Between Jan 2021 – April 2022, burn patients at a single national burn center who needed at least two separate donor sites for split-thickness skin grafting were included. In total, 36 wound pairs in similar anatomical areas were made in 23 patients. Donor sites were infiltrated with tumescence containing epinephrine before harvesting. Paired wounds were randomized to topical application of standard epinephrine solution, with or without added TXA 25 mg/ml, before coverage with vaseline and dry gauze. Endpoints were time to re-epithelialization and surrogate bleeding estimates defined as net weight gain of dressings per cm^2^ wound area, bloodstain area per wound area, and visual rating of the amount of blood in dressings on the first postoperative day.

**Results:**

There was no difference in healing time between wounds. The results trended towards less increase in dressing weight gain per cm^2^ of wound area in the TXA group, though not significant (p=0.12). However, when dividing the results in two at the median bleeding volume, there was a significant reduction in bleeding in the high-bleed group (p=0.012). Similar observations were made for the other two bleeding endpoints, but the differences did not reach statistical significance.

**Conclusions:**

Moistening a superficial wound surface with TXA 25 mg/ml does not impair wound re-epithelialization. Topical TXA may reduce bleeding from graft donor sites with low risk of systemic complications.

**Applicability of Research to Practice:**

This pilot study suggests that topical TXA is of value in reducing bleeding in burn surgery without delaying donor site healing. The effect may be larger in wounds not prepared with epinephrine infiltration. More research is warranted regarding the optimal mode of topical drug delivery and systemic concentrations after topical application in burns. Furthermore, it remains to investigate whether topical TXA affects graft take.

